# Small molecule protein assembly modulators with pan-cancer therapeutic efficacy

**DOI:** 10.1098/rsob.240210

**Published:** 2024-12-18

**Authors:** Anuradha F. Lingappa, Olayemi Akintunde, Erin Samueli, Connie Ewald, Maya Michon, Niloufar Ziari, Ming Lu, Shao Feng Yu, Markus Froehlich, Phuong Uyen Le, Yuniel Fernandez, Suguna Mallesh, Jim Lin, Anatoliy Kitaygorodskyy, Dennis Solas, Jonathan C. Reed, Jaisri R. Lingappa, Andreas Müller-Schiffmann, Carsten Korth, Dharma Prasad, Aysegul Nalca, Emily Aston, Brad Fabbri, Sanjeev K. Anand, Thomas W. Campi, Emma Petrouski, Debendranath Dey, David W. Andrews, James L. Rubenstein, Vishwanath R. Lingappa

**Affiliations:** ^1^ Prosetta Biosciences, San Francisco, CA, USA; ^2^ University of California San Francisco, San Francisco, CA, USA; ^3^ Sunnybrook Research Institute, Toronto, ON, Canada; ^4^ Department of Global Health, University of Washington, Seattle, WA, USA; ^5^ Institute of Neuropathology, Heinrich Heine University, Dusseldorf, Germany; ^6^ United States Army Medical Research Institute for Infectious Diseases, Frederick, MD, USA; ^7^ TechAccel, Overland Park, KS, USA; ^8^ Modulant Biosciences, Fishers, IN, USA

**Keywords:** drug discovery, pan-cancer therapeutics, allosteric modulator, restoration of homeostasis, multi-protein complex

## Abstract

Two structurally unrelated small molecule chemotypes, represented by compounds PAV-617 and PAV-951, with antiviral activity in cell culture against Mpox virus (formerly known as monkeypox virus) and human immunodeficiency virus (HIV) respectively, were studied for anti-cancer efficacy. Each exhibited apparent pan-cancer cytotoxicity with reasonable pharmacokinetics. Non-toxicity is demonstrated in a non-cancer cell line and in mice at doses achieving drug exposure at active concentrations. Anti-tumour properties of both chemotypes were validated in mouse xenografts against A549 human lung cancer and, for one of the chemotypes, against HT-29 colorectal cancer. The targets of these compounds are unconventional: each binds to a different transient, energy-dependent multi-protein complex. Treatment with these compounds alters the target multi-protein complexes in a manner that appears to remove a block, crucial for cancer survival and progression, on a homeostatic linkage between uncontrolled proliferation and apoptosis. These compounds provide starting points for development of novel, next-generation, non-toxic, pan-cancer therapeutics.

## 1. Background

The similarity of the interactions of viral infection and cancer with the healthy host has often been noted [1]. Both represent pathological processes of extremely diverse origin, that overcome the complex feedback controls of homeostasis, to the detriment of the host [2]. Both exploit natural selection as a powerful weapon to overcome host defences. Viruses have done so over deep evolutionary time through co-evolution with their hosts and through the emergence of resistance mutations in response to the selective pressure of treatments [3,4]. Cancers regularly use the latter mechanism in real time, resulting in clonal mutants that drive cancer progression and chemotherapy resistance [5].

Viruses representing at least seven different viral families—Epstein-Barr virus (EBV), hepatitis B virus (HBV), hepatitis C virus (HCV), human T-lymphotropic virus 1 (HTLV-1), human papillomavirus (HPV), Kaposi sarcoma-associated herpesvirus (KHSV or HHV-8) and Merkel cell polyomavirus (MCPyV)—are known to be directly oncogenic through integration into the genome, alteration of the cellular micro-environment and/or impairment of the host’s innate immune system defences [6,7]. It has therefore been proposed that viruses could play a key role in the discovery of new cancer treatments by identifying cellular targets that drive tumourigenesis [8,9].

The incompleteness in our current understanding of the dynamics of host homeostasis and its myriad of feedback controls has been a disadvantage for efforts to design novel therapeutic countermeasures against both viruses and cancer. However, a recent unconventional approach to antiviral drug discovery has identified small molecules targeting hard-to-detect allosteric sites essential for host homeostasis, that appear to be repurposed upon viral infection, and restored upon drug treatment [10]. We wished to determine whether a subset of these compounds might have therapeutic applicability against cancer, given the analogy that both viruses and cancers drive departure from homeostasis. The results to be presented here suggest this is the case and shed light on novel molecular pathways relevant for both viral and neoplastic disease, providing a strategy for development of novel cancer therapeutics.

Using a cell-free protein synthesis and assembly (CFPSA) system, compounds from a library of approximately 150 000 drug-like small molecules were screened for hits which blocked the host-catalysed assembly of viral capsids without inhibiting protein synthesis [10–13]. Hit compounds from this screen have been termed ‘protein assembly modulators’ [10]. A collection of 300 structurally diverse protein assembly modulators have been validated against infectious virus in cell culture for one or more viral families including Retroviridae, Rhabdoviridae, Filoviridae, Arenaviridae, Bunyaviridae, Flaviviridae, Poxviridae, Adenoviridae, Herpesviridae, Paramyxoviridae, Coronaviridae, Orthomyxoviridae and Picornaviridae [10,12–15]. In a number of cases, including Coronaviridae and Paramyxoviridae, cellular activity has been confirmed in animal disease models [10].

These antiviral protein assembly modulators appear to disrupt host–viral protein–protein interactions via allosteric sites that control repurposing of host assembly machinery for viral capsid formation and which also allow disengagement of host innate immune defences such as autophagy [10]. In an earlier study, a class of protein assembly modulators was shown to change the composition of a transient, energy-dependent multi-protein complex whose components include p62/SQSTM1, a key regulator of autophagy [10]. Upon treatment with that antiviral assembly modulator, the target multi-protein complex is restored to its composition in uninfected cells, with the loss of the viral nucleoprotein and restoration of p62/SQSTM1 [10].

The discovery of such dynamic multi-protein complexes whose composition changes with drug treatment offers a new means of parsing post-translational protein heterogeneity and its relevance for diseased states. Remarkably, the amount of the proteins observed in these transient multi-protein complexes targeted by antiviral assembly modulators comprise only a very small fraction of the total amount of those proteins present in a cell [10,12]. Moreover, once depleted, no further binding to drug resins occur, indicating that the small fraction that form these drug targets and bind to the drug resin, is not in equilibrium with the balance that does not. The role played by the subset of the protein that is part of a particular multi-protein complex may reflect a ‘moonlighting’ function, as observed for a growing number of cellular and viral proteins [16–18].

We hypothesized that if an overlap between viral and oncogenic pathways exists, some antiviral assembly modulators might be capable of disrupting a related multi-protein complex associated with uncontrolled proliferation, an iconic hallmark of cancer [19,20]. To test the hypothesis, we established a cancer-relevant counter screen and applied it to the collection of previously identified antiviral protein assembly modulator compounds. In this paper, we will describe two protein assembly modulators which were originally characterized for their antiviral properties but are now shown to have potential as cancer therapeutics based on cellular screening and animal validation studies. Just as the previously cited study discovered a pan-respiratory antiviral chemotype that appears to target a host–viral interface, so also we hypothesized that it should be possible to find a comparable anti-cancer chemotype that reverses changes selected by diverse cancers that allow them to escape from feedback constraints that normally prevent uncontrolled proliferation.

## 2. Results

### 2.1. Uncontrolled cellular proliferation: a hallmark of cancer inhibited by selected protein assembly modulators

No hallmark of cancer is more fundamental than uncontrolled proliferation [19]. Uncontrolled proliferation normally triggers cell death mechanisms, including apoptosis [21]. Therefore, to survive, a cancer must achieve a means of evading cell death long enough to complete cell division and reset the cell death timer. This, in turn, allows further proliferation, during which time additional mutations can occur and selection pressure will drive higher and higher grade malignancy and so on, ultimately resulting in metastasis [22]. The regulatory feedback loops that detect uncontrolled proliferation and direct such cells to apoptosis remain incompletely understood. If cancers can emerge when those mechanisms are impaired, it seemed plausible to us that the previously identified collection of antiviral assembly modulators might include some chemotypes that restore relevant feedback loops by antagonizing the changes selected by cancers for their survival. One such phenotype would be arrest of cells engaging in such uncontrolled proliferation, allowing endogenous apoptotic mechanism to proceed in a timely manner. Compounds capable of arresting proliferation, either directly or indirectly, would make potent anti-cancer agents, especially if cytotoxicity is selective for cancer cells or if the delay in cancer progression would provide an opportunity for a patient’s innate immune system and other homeostatic feedback loops to re-establish themselves, and drive cancer into apoptosis.

Abnormal signalling pathways triggered by aberrant protein–protein interactions is one way that neoplastic cells are known to able to achieve uncontrolled proliferation [22]. In order to characterize whether assembly modulators could selectively arrest the proliferation of neoplastic cells by redirecting key protein–protein interactions, we first sought to identify a cell line in which endogenous apoptosis was substantially lacking. While a successful anti-cancer compound would likely exhibit both anti-proliferative and cytotoxic efficacy, we wanted to conduct our screen under conditions where a readout measuring the arrest of proliferation would not be obscured by downstream activation of the normal cascade of events comprising cell death pathways. In this way, compounds efficacious by the desired mechanism would be distinguished from those that are more conventionally cytotoxic.

**Figure 1.**
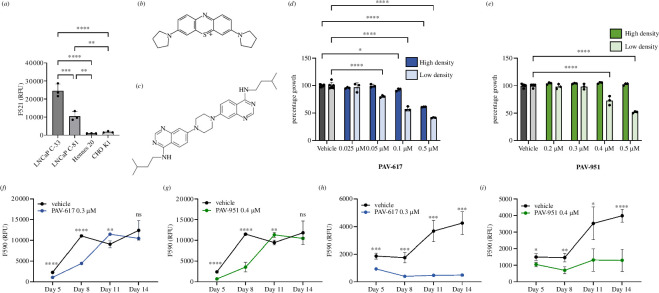
Assay development and activity of hit compounds PAV-617 and PAV-951. (*a*) Assessment of the endogenous apoptosis response in multiple cell lines. Plates were seeded with LNCaP C-33, LNCaP C-81, CHO K-1 and Hennes 20 cells. After 3 days of growth Apo-ONE reagent was added and caspase-3/7 activity was determined by fluorescent readout. Averages, standard deviation of observed activity in triplicate-repeated samples and *p*-values using an ordinary one-way ANOVA test were calculated and graphed in GraphPad Prism. The Hennes 20 cell line was chosen for the counterscreen based on its low levels of caspase activity. (*b*) The disclosed chemical structure of PAV-617, one hit compound from the Hennes 20 screen. (*c*) The disclosed chemical structure of PAV-951, a second hit compound from the Hennes 20 screen. (*d,e*) Activity of (*d*) PAV-617 and (*e*) PAV-951 in the Hennes 20 ‘arrest of proliferation’ assay where parallel plates of Hennes 20 cells were seeded at a high density of 15 000 cells per well and a low density of 500 cells per well and treated with DMSO, PAV-617 or PAV-951. Fluorescent reading (RFU) corresponding to cell viability was calculated using an AlamarBlue assay and the averages, standard deviations for triplicate-repeated samples and *p*-values using a two-way ANOVA test were calculated and graphed on GraphPad Prism as a percentage of the DMSO-treated cells. (*f–i*) Recovery of cancer cell growth following removal of compound. (*f–g*) Hennes 20 or (*h–i*) LNCaP C-33 cells were seeded at a low density and then incubated with DMSO, PAV-617 or PAV-951. After a period of treatment, the medium containing compound was removed and replaced with fresh media. Plates were assessed for cell viability by AlamarBlue on day 5, 8, 11 and 14 and the averages, standard deviations of triplicate-repeat samples, and *p*-value using unpaired *t*-tests were calculated and graphed over time on GraphPad Prism. PAV-617 and PAV-951 treated Hennes 20 and LNCaP C-33 cells all showed reduced viability compared with matched DMSO-treated cells on day 5. However, cell growth in the Hennes 20 cells which had been treated with compound recovered over time, while the LNCaP C-33 cells which had been treated with compound did not recover.

We assessed caspase-3/7 activity in multiple tumour cell lines with an Apo-ONE assay (see figure 1*a*). The expected correlation between endogenous triggers of apoptotic death and cancer progression was demonstrated in the LNCaP prostate cancer progression cell model, where LNCaP C-33 early (hormone sensitive) cancer cells displayed substantially more markers of apoptosis than LNCaP C-81 late (hormone resistant) cancer cells (see figure 1*a*) [23]. In the Apo-ONE assay, CHO K-1 cells show very little endogenous apoptosis (see figure 1*a*). Hennes 20, a CHO K-1 derivative into which the human APP gene has been transfected, show even less apoptosis than their parental line (see figure 1*a*). We were interested in using a cell line with this phenotype, irrespective of its precise molecular basis.

Our collection of antiviral assembly modulator compounds were then counter screened in Hennes 20 cells plated at low (500 cells per well) versus high (15 000 cells per well) densities and treated with DMSO (vehicle) or dose-titration of compounds. The rationale for this screen is that an intrinsically toxic compound should kill cells regardless of cell density, including in Hennes 20 cells. Thus, when the readout is a cell viability assay such as AlamarBlue, a compound that selectively triggers the arrest of proliferation will appear cytotoxic due to inhibition of cell growth when plated at low density followed by 72 h to proliferate in the absence versus presence of drug. However the same compound will appear non-toxic to the same cells plated at a high density approaching confluence, since the cell density upon arrest is already close to the maximum.

Two structurally unrelated small molecules, PAV-617 and PAV-951 (Tanimoto similarity score of 41%), displayed the desired phenotype of a difference between their inhibitory effects on low and high density-plated Hennes 20 cells (see figure 1*b*,*c* for disclosed chemical structures, electronic supplementary material, figures S1A and S2A, for synthetic schemes, and figure 1*d*,*e* for their activity in the low versus high cell density Hennes 20 screen).

The results at low versus high density in Hennes cells suggested that the effect of these compounds in cells lacking endogenous apoptosis was due to inhibition of proliferation. To confirm that the inhibition observed in low-density Hennes 20 cells resulted from temporary arrest of proliferation and not cell death, compound was removed after a period of treatment and cell growth was measured over the subsequent two weeks for recovery potential. Once compound was removed, following a period of recovery, cell proliferation was restored over time for both chemotypes (see figure 1*f*,*g*). By day 11, cell density in compound-treated cells had caught up to that in the DMSO-treated cells (see figure 1*f*,*g*).

Since Hennes 20 cells do not appear to have endogenous apoptosis, we sought to use a different cancer cell line with substantial endogenous apoptosis to assess whether that inhibition of proliferation would be accompanied by cell death. The recovery experiment was repeated in LNCaP C-33 cells which have substantial endogenous apoptosis (see figure 1*h*,*i*). Treatment with PAV-617 and PAV-951 inhibited LNCaP C-33 growth relative to the DMSO-treated control, but the cells did not recover or grow significantly once compound was removed (see figure 1*h*,*i*). This finding supports our hypothesis that the action of our compounds is primarily on arrest of proliferation and that only in a cancer line that maintains endogenous apoptosis, does growth arrest progress to death.

### 2.2. Investigating the activities of PAV-617 and PAV-951: from modulators of viral capsid assembly to pan-cancer therapeutics

**Figure 2.**
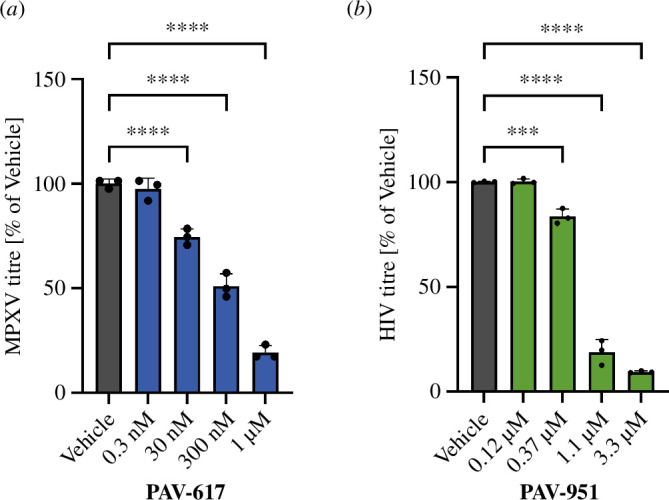
Antiviral properties of PAV-617 and PAV-951. (*a*) Activity of PAV-617 against Mpox. BSC-40 cells were infected with 100 plaque forming units of Mpox Zaire 79 and treated with PAV-617 for 3 days. Averages and standard deviation for plaques observed with triplicate-repeated dose-titration of PAV are shown as a percentage of the plaques observed in untreated cells. Statistical significance calculated on GraphPad Prism using an ordinary one-way ANOVA test is indicated on the graph by asterisks. (*b*) Activity of PAV-951 against HIV. MT-2 cells were infected with NL4-3 Rluc HIV and treated with PAV-951 for 4 days. Averages and standard deviation of viral titer observed with triplicate-repeated dose-titration of PAV-951 are shown as a percentage of the titer observed in DMSO-treated cells. Statistical significance calculated on GraphPad Prism using an ordinary one-way ANOVA test is indicated on the graph by asterisks.

The anti-proliferative compounds PAV-617 and PAV-951 had originally emerged from our CFPSA screen as inhibitors of viral capsid formation. The CFPSA model has been validated by demonstrating that antiviral assembly modulator hits display activity against infectious viruses in cell culture [10,12–15]. PAV-617 is active against pox viruses in cell culture [14]. The effective concentration for half maximal activity (EC50) of PAV-617 against Mpox was 201.4 nM (see figure 2*a*). PAV-951 is active against HIV in cell culture. The EC50 of PAV-951 against HIV was 660 nM (see figure 2*b*).

When PAV-617 and PAV-951 were identified as having anti-cancer activity in addition to antiviral properties, we suspected that the compounds might correct cancer-induced defects in protein assembly that were related to the virally induced aberrant assembly pathways which we had been studying. We sought to better understand how the defects present themselves across diverse cancers by screening the compounds in as many different cancers cell lines (derived from a variety of tissues and representative of male and female patients of different ethnicities and ages ranging from paediatric to senior) that we could. We conducted an in-house screen against four cancers (A549, HT-29, LNCaP C-33 and PANC-1). We also sent the compounds for more extensive assessment in the National Cancer Institute 60 cell line screen (NCI-60), contract research organizations (CROs) and in the labs of academic collaborators.

**Figure 3.**
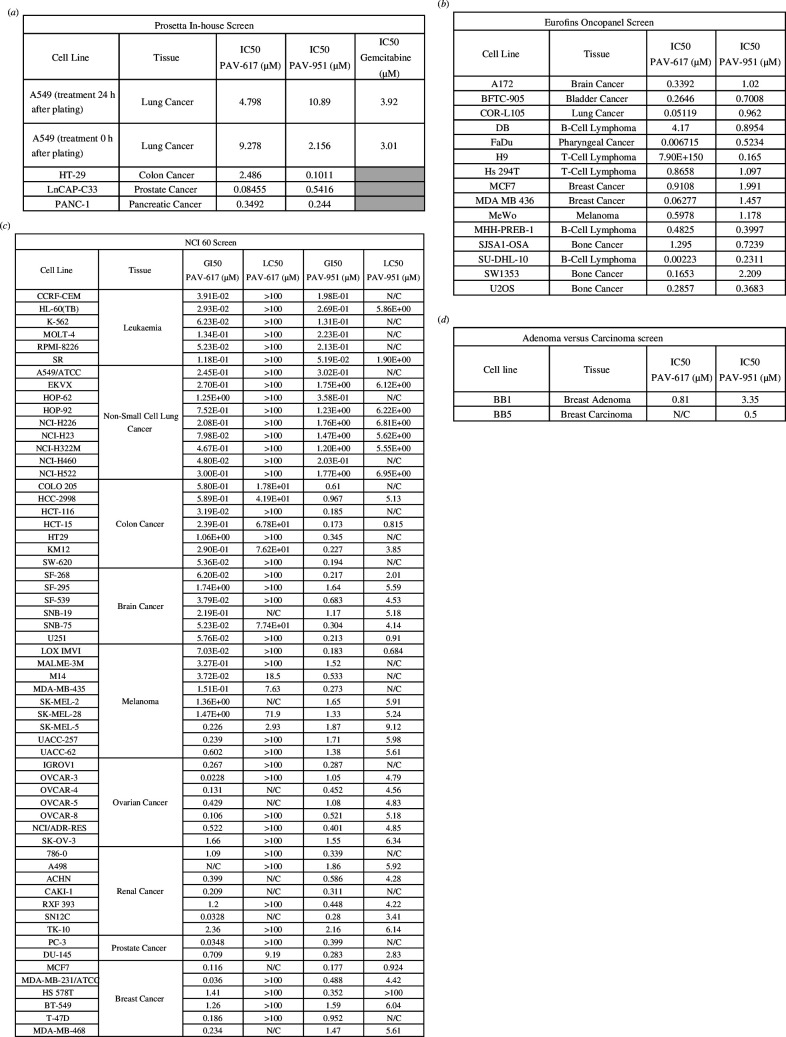
Pan-cancer activity of PAV-617 and PAV-951 as assessed by four methodologies. (*a*) The IC50s of PAV-617 and PAV-951 assessed in house against A549, HT29, LNCaP C-33 and PANC-1 cell lines. (*b*) The IC50s of PAV-617 and PAV-951 in the Eurofins OncoPanel against A172, BFTC-905, COR-L105, DB, FaDu, H9, Hs 294T, MCF7, MDA MB 436, MeWo, MHH-PREB-1, SJSA1-OSA, SW1353 and U2OS cell lines. (*c*) The GI50 and the LC50 of PAV-617 and PAV-951 in the NCI-60 screen against CCRF-CEM, HL-60 (TB), K-562, MOLT-4, RPMI-8226, SR, A549/ATCC, EKVX, HOP-62, HOP-92, NCI-H226, NCI-H23, NCI-H322M, NCI-H460, NCI-H522, COLO 205, HCC-2998, HCT-116, HCT-15, HT29, KM12, SW-620, SF-268, SF-295, SF-539, SNB-19, SNB-75, U251, LOX IMVI, MALME-3M, M14, MDA-MB−435, SK-MEL-2, SK-MEL-28, SK-MEL-5, UACC-257, UACC-62, IGROV1, OVCAR-3, OVCAR-4, OVCAR-5, OVCAR-8, NCI/ADR-RES, SK-OV-3, 786-0, A498, ACHN, CAKI-1, RXF 393, SN12C, TK-10, PC-3, DU-145, MCF7, MDA-MB-231/ATCC, HS 578T, BT-549, T-47D, MDA-MB-468 cell lines. (*d*) The IC50 against BB1 and BB5 patient biopsy derived cell lines. N/C indicates not calculated.

The standard approach to cell culture assessment of compound potency against cancer cell lines is to trypsinize cells to release them from the source plate, re-plate them, wait 24 h for their recovery and then treat with drug, measuring the effect of the drug over the subsequent 24 to 72 h [24]. In our initial in-house screen, cells were plated and treated with DMSO (vehicle) or a dose-titration of compound after 24 h. The cells were incubated with vehicle or compound for an additional 72 h then analysed by AlamarBlue, to compare cell viability of the DMSO-treated and compound-treated groups. Both PAV-617 and PAV-951 displayed activity against all four cancers (see figure 3*a* for IC50s and electronic supplementary material, figures S3 and S4, for dose-titrations).

Later, the protocol was adapted to one where compound was added immediately following trypsinization and re-plating of cells, and incubation continued for the same period of time. The change in timing of re-plating and drug treatment did not significantly affect the IC50 of Gemcitabine hydrochloride, which was assessed as a conventional anti-cancer positive control (see figure 3*a* for IC50s and electronic supplementary material, figures S3, S4 and S9, for dose-titrations of PAV-617 PAV-951 and Gemcitabine). However, the sensitivity of A549 to PAV-951 increased significantly when added concurrently with plating (see figure 3*a* for IC50s and electronic supplementary material, figures S4 and S9).

In the Eurofins OncoPanel, 15 cancer cell lines (A172, BFTC-905, COR-L105, DB, FaDu, H9, Hs 294T, MCF7, MDA MB 436, MeWo, MHH-PREB-1, SJSA1-OSA, SW1353 and U2OS) were plated and treated with DMSO (vehicle) or a dose-titration of compound after 24 h. The cells were incubated for an additional 3 days then analysed by CellTiter-Glo, to compare cell viability of the DMSO-treated and compound-treated groups. Both PAV-617 and PAV-951 displayed inhibitory activity against all 15 cancers, as well as against the two non-cancerous cell lines (see figure 3*b* for IC50s and electronic supplementary material, figures S3 and S4, for dose-titrations).

In the NCI-60 screen, 59 cancer cell lines (CCRF-CEM, HL-60 (TB), K-562, MOLT-4, RPMI-8226, SR, A549/ATCC, EKVX, HOP-62, HOP-92, NCI-H226, NCI-H23, NCI-H322M, NCI-H460, NCI-H522, COLO 205, HCC-2998, HCT-116, HCT-15, HT29, KM12, SW-620, SF-268, SF-295, SF-539, SNB-19, SNB-75, U251, LOX IMVI, MALME-3M, M14, MDA-MB-435, SK-MEL-2, SK-MEL-28, SK-MEL-5, UACC-257, UACC-62, IGROV1, OVCAR-3, OVCAR-4, OVCAR-5, OVCAR-8, NCI/ADR-RES, SK-OV-3, 786-0, A498, ACHN, CAKI-1, RXF 393, SN12C, TK-10, PC-3, DU-145, MCF7, MDA-MB-231/ATCC, HS 578T, BT-549, T-47D, MDA-MB-468) were grown for 24 h then treated with vehicle or compound, or fixed *in situ* with TCA, to represent a measurement of the cell population for each cell line at the time of drug addition. The treated cells were grown for an additional 48 h before being fixed *in situ* with TCA. Fixed cells were then stained with Sulforhodamine B. Absorbance was read to determine cell viability of compound-treated cells relative to both the time at which treatment began and to untreated cells at the end of the study. The NCI-60 screen was initially conducted at a single dose of 2.5 µM, but was subsequently repeated with a dose-titration. From these readings, separate growth inhibition of 50% (GI50) and lethal inhibition of 50% (LC50) values could be calculated, depending on if the measured protein remaining after compound-treatment was less than the measured protein in control-treated cells and/or the measured protein in cells at the time compound was added.

PAV-617 and PAV-951 both showed inhibition of growth against all cancers assessed in the NCI-60 screen (see figure 3*c* for IC50s and electronic supplementary material, figures S3 and S4, for dose-titrations). However, PAV-617 did not show lethality on most cell lines even when assessed at the highest dose of 100 µM (see figure 3*c* for IC50s and electronic supplementary material, figures S3 and S4, for dose-titrations). The delta between the dose where PAV-617 inhibited growth relative to vehicle-treated cells (in the 2–3 digit nanomolar range for more than 40 cells lines) and the dose required to decrease cell viability below what was measured at the time compound was added (>100 µM for at least 40 cell lines) was striking. By contrast, PAV-951 did show lethality on many of the cancer cell lines, though for most cell lines a significantly higher dose of PAV-951 was required to achieve lethality compared with the dose needed to achieve growth inhibition (see figure 3*c* for IC50s and electronic supplementary material, figures S3 and S4, for dose-titrations).

Two cell lines, BB1 and BB5, were derived from two patient breast biopsies that were obtained prior to treatment. Follow up classification indicated that BB1 was a benign adenoma and BB5 was a breast adenocarcinoma. The cells were grown in conditional reprogramming media in co-culture with irradiated fibroblasts. Once cultures reached exponential growth phase, the cells were harvested and seeded. After 48 h of growth, cells were treated with PAV-617, PAV-951, 0.1% DMSO (vehicle) or 1 µM staurosporine (positive control which induced cell death) for 60 h. Compound-treated cells were imaged with Hoechst 33342, TMRE and ChromaLive which stained the nucleus, functional mitochondria and cellular membranes, respectively. Cells were imaged by confocal fluorescence microscopy and IC50s were calculated as percent ChromaLive negative (see figure 3*d* for IC50s and electronic supplementary material, figure S5, for dose titrations and images of DMSO compared with PAV-951-treated BB1 and BB5 cells). PAV-617 and PAV-951 had higher IC50s against the BB1 adenoma (0.81 µM and 3.35 µM, respectively) than was observed against cancerous cell lines including the BB5 carcinoma (see figure 3*d* and electronic supplementary material, figure S5).

The four screens described above each measure anti-cancer properties by different metrics. PAV-617 and PAV-951 demonstrated activity by all four methods, indicating replicability of the data. Between the four screens, both compounds exhibited inhibition of over 70 different cancers, representative of bladder cancer, bone cancer, brain cancer, breast cancer, colon cancer, lung cancer, leukaemia, B cell and T cell lymphoma, melanoma, ovarian cancer, pancreatic cancer, pharyngeal cancer, prostate cancer and renal cancer (see figure 3 and electronic supplementary material, figures S3–S5).

### 2.3. Animal validation of PAV-617 and PAV-951 anti-tumour efficacy at non-toxic doses

With demonstrated antiviral activity, demonstrated anti-cancer activity and data supporting a proliferation based mechanism-of-action, we assessed mouse toxicology and pharmacokinetic (PK) properties in order to determine suitability for efficacy studies in animal models.

The maximum tolerated dose (MTD) estimates how much compound can be administered to an animal without adverse effects [25]. When administered by intraperitoneal (IP) injection, PAV-617 was found to be safe at 15 mg kg^−1^ and PAV-951 was safe at 2.5 mg kg^−1^ (see electronic supplementary material, figure S6A–C for body weight and haematology of male and female Balb/c mice following 10 days of daily dosing with 2.5 mg kg^−1^ PAV-951). The PK properties are based on the absorption, distribution, metabolism and excretion of a compound in a living organism and are necessary to determine dosing parameters because any compound designed for clinical use needs to achieve an efficacious concentration in a target organ [26,27]. As an early measure of PK, we determined the concentration of compound in the plasma and lungs of mice or rats over time following one intravenous (IV) or IP dose (see electronic supplementary material, figure S6D–F for PAV-617 and PAV-951 PK).

We determined from the PK data that, while both chemical series would need optimization on PK and toxicological properties before being named as clinical drug-candidates, PAV-617 and PAV-951 achieved sufficient exposure in the lung that they would be adequate for a preliminary animal efficacy study in order to validate whether or not the anti-proliferative properties of the compound observed in cell culture translates to efficacy in animals.

In the first set of animal efficacy studies, human A549 non-small cell lung cancer cells were grafted subcutaneously onto mice. After 30 days of tumour establishment, the animals received daily treatment with PAV-617 or PAV-951 and tumour volume was measured over time. The doses and routes of administration for PAV-617 (10 mg kg^−1^ IP injection) and PAV-951 (1.5 mg kg^−1^ IV injection) were determined based on their MTD and PK properties. The PAV-617 study was conducted for 28 days and the PAV-951 study was conducted for 14 days. As negative and positive controls, both studies included a group treated with vehicle only and a group treated with Gemcitabine hydrochloride, an FDA approved drug for non-small cell lung cancer, administered to animals in the same way as the test compound.

**Figure 4.**
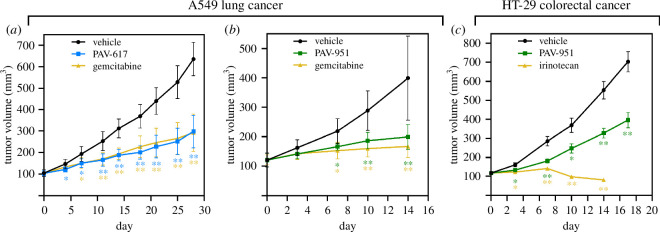
Efficacy of PAV-617 and PAV-951 in animal cancer xenografts. Tumours were grafted onto mice via a subcutaneous injection and grown until they reached a volume of 100 mm^3^. Animals were divided into randomized groups and treated with vehicle, PAV-617, PAV-951 or an FDA approved cancer drug as a positive control. (*a*) Tumour volume across time for an A549 lung cancer treated with vehicle, PAV-617 or Gemcitabine hydrochloride. (*b*) Tumour volume across time for a A549 lung cancer treated with vehicle, PAV-951 or Gemcitabine hydrochloride. (*c*) Tumour volume across time for a HT-29 colorectal cancer treated with vehicle, PAV-951 or Irinotecan.

The Gemcitabine was administered at the standard dose of 100 mg kg^−1^. Both PAV-617 and PAV-951 reduced tumour growth significantly compared with the vehicle-only groups and performed comparably with Gemcitabine despite being administered at substantially lower doses. PAV-617 displayed a tumour growth inhibition (TGI) of 63% compared with Gemcitabine’s TGI of 64% while PAV-951 had a TGI of 72% compared with Gemcitabine’s TGI of 84% (see figure 4*a*,*b*). Survival was not assessed as part of the study.

In the second animal efficacy study, human HT-29 colorectal adenocarcinoma cells were grafted subcutaneously onto mice. After tumour establishment, the animals received treatment with vehicle, 3 mg kg^−1^ PAV-951 or 60 mg kg^−1^ of a positive control drug Irinotecan. After 17 days of treatment, PAV-951 had significantly reduced tumour growth relative to the vehicle-only group (TGI of 52%), though the impact was lower than Irinotecan (TGI of 108%) (see figure 4*c*). Survival was not assessed as part of the study.

### 2.4. Characterizing the molecular targets of PAV-617 and PAV-951

As PAV-617 and PAV-951 were identified by phenotypic screens, their actual targets were unknown during the early stages of compound advancement. To identify their targets, each molecule was coupled to affi-gel resins from a position on the molecule unrelated to proliferation arrest activity (see electronic supplementary material, figure S1A and B for synthetic schemes of resins). In that way, they could serve as target-bindings ligands for drug resin affinity chromatography (DRAC) [28]. A549 lung cancer was chosen for DRAC starting material as both PAV-617 and PAV-951 had shown efficacy against A549 in our screen, the NCI-60 screen and the mouse xenograft model.

Extracts were prepared from A549 cells and applied to the resins. The resins were then washed with 100 bed volumes of buffer and eluted overnight with either 100 µM PAV-617 or 100 µM PAV-951. In view of the previous demonstration of energy-dependent stimulation of target engagement by assembly modulators [10], the DRAC experiments were conducted in parallel under ‘cold’ conditions (at 4°C in the absence of added nucleotide triphosphates) and ‘hot’ conditions (at room temperature, and where both the starting extract and the eluate were spiked with an ‘energy cocktail’ of ribonucleotide triphosphates (1 mM rATP, 1 mM rGTP, 1 mM rCTP, 1 mM UTP) 4mM creatine phosphate and 5 µg ml^−1^ creatine kinase (final concentrations) for energy regeneration. For each starting material/compound condition, we also eluted from a negative control resin which comprised an affi-gel matrix without coupled drug ligand.

**Figure 5.**
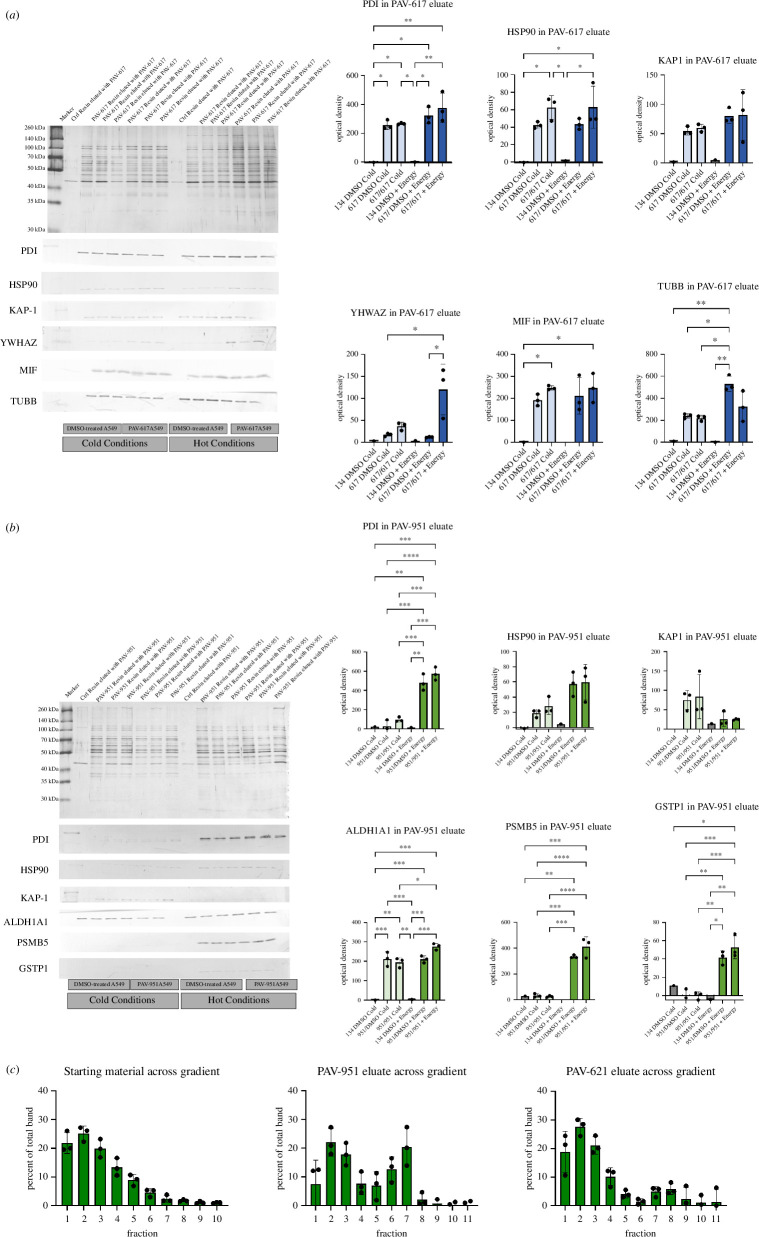
DRAC of PAV-617 and PAV-51 reveal dynamic multi-protein complex targets. Drug resin affinity chromatography experiments, as described in §4 were carried out with PAV-617 and PAV-951 as well as a negative control resin (134). (*a*) Analysis of PAV-617 resin eluate under hot and cold conditions by silver stain and western blot for PDI, HSP90, KAP-1, YWHAZ, MIF and TUBB with pictures of the western blot underneath the silver stain. The protein band detected by western blot was quantified (graphs of the quantified bands are shown to the right) and statistical significance in different amounts of protein detected in the eluate under different conditions is denoted by asterisks. (*b*) Analysis of PAV-951 resin eluate under hot and cold conditions by silver stain and western blot for PDI, HSP90, KAP-1, ALDH1A1, PSMB5 and GSTP1 with pictures of the western blot underneath the silver stain. The protein band detected by western blot was quantified (graphs of the quantified bands are shown to the right) and statistical significance in different amounts of protein detected in the eluate under different conditions is denoted by asterisks. (*c*) The distribution of the protein ALDH1A1 across a sucrose gradient from starting A549 extract as well as PAV-951 and PAV-621 resin free drug eluate. For each condition, a representative western blot for ALDH1 from triplicate-repeated gradients are pasted below quantitation of the average distribution of the protein across the gradient, with error bars indicating standard deviation. The quantitation shows amount of ALDH1A1 in each particular fraction as a percent of the total ALDH1A1 found across all fractions.

When the eluates were analysed by silver-stained SDS–PAGE gels, they appeared to contain sets of proteins (see figure 5*a*,*b* for silver stains of PAV-617 and PAV-951 resin eluates in triplicate). DRAC Eluates from DMSO-treated starting material were sent for analysis by tandem mass spectrometry (MS-MS). For PAV-617, eluates conducted under cold versus hot conditions were sent for MSMS as well as control eluates generated from a negative control which comprised an affi-gel matrix without coupled drug ligand.

A total of 187 proteins were identified by MSMS in the PAV-617 resin eluate under cold conditions and 74 under hot conditions, after subtraction of spectral counts detected in the control resin eluate. After subtraction, 135 proteins were only found in cold conditions, 2 were only in hot conditions and 52 were found in both. The protein Macrophage Migration Inhibitory Factor (MIF), which has previously been identified as a target of PAV-617, was present under hot conditions [29]. Sixty-three of the identified proteins were known oncogenes in the Bushman labs oncogene database (http://www.bushmanlab.org/links/genelists) and 14 of the identified proteins were associated with the HIV interactome as described by Jager *et al.* [30] (see electronic supplementary material, figure S7A).

HSP90, MIF, PDI, TUBB and YWHAZ, which had been detected in the PAV-617 drug resin eluate by MSMS, was confirmed by western blot of triplicate repeated eluate samples (see figure 5*a*). KAP1 (also known as TRIM28) was identified in the PAV-617 eluate by western blot even though it was not identified by MSMS (see figure 5*a* and electronic supplementary material, figure S7A). By western blot, the amount of HSP90, TUBB and YWHAZ in the PAV-617 resin eluate increased under hot conditions (see figure 5*a*).

Two hundred and fourteen proteins were identified in the PAV-951 resin eluate after subtraction of spectral counts detected in the control resin eluate. Eighty-seven of those proteins were present in triplicates (see electronic supplementary material, figure 7B). Twenty-nine proteins from the PAV-951 eluate triplicates were found to be oncogenes in the Bushman labs oncogene database (http://www.bushmanlab.org/links/genelists) and 13 were associated with the HIV interactome as described by Jager *et al.* (see electronic supplementary material, figure S7B) [30]. The protein identified by spectral count in largest amounts in the PAV-951 resin/eluate was Aldehyde dehydrogenase (ALDH1A1), a protein implicated in proliferation of stem-like cells and poor prognosis following the progression to metastasis and development of drug resistance [31–35].

HSP90, PDI, ALDH1A1, PSMB5 and GSTP1, which had been detected in the PAV-951 drug resin eluate by MSMS, were confirmed by western blot of triplicate repeated samples (see figure 5*b*). KAP1 (also known as TRIM28) was identified in the PAV-951 resin eluate by western blot even though it was not identified in the MSMS (see figure 5*b* and electronic supplementary material, figure S7B). The amount of PSMB5, PDI and GSTP1 increased under hot conditions, while the amount of KAP1 in the PAV-951 resin eluate decreased under hot conditions (see figure 5*b*).

The DRAC experiments under both cold and hot conditions were conducted in parallel with starting extract derived from A549 cells treated for 20 h with 500 nM PAV-617, 500 nM PAV-951 or vehicle. The eluates of compound-treated starting material appeared enriched for selected proteins (see figure 5*a–c*). The eluate composition for both chemotypes appeared to change in response to the variables of both compound-treatment of the cells and the presence of metabolic energy during DRAC.

One explanation for why the DRAC eluates contain large, dynamic sets of proteins is that the targets for PAV-617 and PAV-951 are themselves multi-protein complexes, as had been observed for analogous studies on structurally unrelated protein assembly modulators effective in other therapeutic areas [10,12]. To test this hypothesis and determine if the proteins found in the eluate were physically together, PAV-951 resin eluates were run across sucrose gradients. In PAV-951 resin eluate, ALDH1A1 was found to run as two peaks, one in fraction 2, and another in fraction 7 (see figure 5*c*). The distribution of ALDH1A1 across the gradient from the eluate was distinctly different from its distribution in the starting material run across a gradient, where ample material was observed in fraction 1 (see figure 5*c*). This finding is consistent with the hypothesis that ALDH1A1 is found in at least two multi-protein complexes that are targeted by PAV-951.

### 2.5. Structure–activity relationship (SAR) exploration of the PAV-951 chemical series

Novel chemical analogs were synthesized based on PAV-951 and analysed by dose-titration from 100 nM to 12.5 µM in our in-house screen and shown to display a robust structure–activity relationship (SAR).

**Figure 6.**
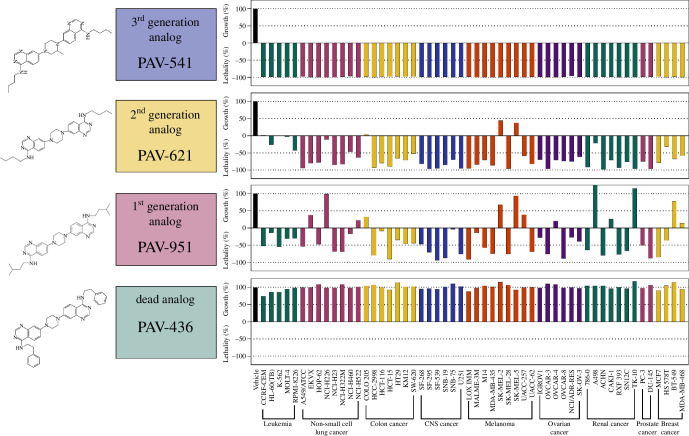
SAR exploration of the PAV-951 series. Chemical analogs were made of PAV-951 and assessed in the NCI-60 screen. Chemical structures of select PAV-951 analogs are disclosed to the left, and their activity against 54 cancers in the NCI-60 cell line screen is to the right. The full 60 panel was not tested on all analogues. The NCI-60 data is plotted where cell viability upon drug treatment is shown relative to the vehicle-treated control and the number of cells at time 0. Values between 0 and 100 represent percent growth inhibition. Values between 0 and negative 100 represent percent cellular lethality.

Activity of selected analogs were confirmed by the NCI-60 screen. Single-dose testing conducted at 2.5 µM confirm that some analogs, such as PAV-436, lost all anti-cancer activity on all cell lines examined (see figure 6). SAR exploration also led to the identification of compounds such as PAV-621 and PAV-541 which showed improvements relative to PAV-951 in the NCI-60 screen with regards to both the breadth of pan-cancer activity and to the metric of lethality to cancers, rather than growth inhibition (see figure 6).

As had been observed with PAV-951, the advanced analog PAV-621 is selectively toxic to the A549 cancer line compared with non-cancer MRC-5 cells (see electronic supplementary material, figure S6). Similarly PAV-621 and PAV-541, but not Gemcitabine, showed the increased potency observed for PAV-951, when added at the time of plating, rather than 24 h later. (see electronic supplementary material, figure S10).

Mouse safety was assessed for PAV-621 and PAV-541 and both were found to be safer than PAV-951. PAV-621 was safe at an IP dose of 7.5 mg kg^−1^. PAV-541 was safe at an IP dose of 5 mg kg^−1^. Mouse PK of PAV-621 showed comparable exposure with PAV-951 in the lung (see electronic supplementary material, figure S7G, for a comparison of PAV-951 and PAV-621 PK).

PAV-621 was coupled to a resin and PAV-621 resin eluate was compared with PAV-951 resin eluate. By silver stain, PAV-621 resin eluate contained a set of proteins which appeared very similar to the PAV-951 resin eluate components (see electronic supplementary material, figure S9A). By MSMS, the eluate of PAV-621 contained 64 proteins, and all but two were also detected in at least one PAV-951 eluate sample by MSMS (see electronic supplementary material, figure S8B). The amount of ALDH1A1 appeared relatively similar between the PAV-951 and PAV-621 resin eluates, but other proteins that had been identified in the PAV-951 resin eluate such as PDI, PSMB5 and GSTP1 were either missing or substantially diminished in the PAV-621 resin eluate (see electronic supplementary material, figures S8B and S9B).

A549 starting extract was applied to the PAV-951 or PAV-621 or control resins and then the depleted flow throughs were put back onto a second copy of the same resin, washed and stripped from the resin with 1% SDS. Western blot for ALDH1A1 indicated that both PAV-951 and PAV-621 resins depleted the extract of targetable ALDH1A1 (see electronic supplementary material, figure S9B).

Compared with PAV-951, when PAV-621 resin eluate was run on a sucrose gradient, substantially more ALDH1A1 appeared in the fraction 2 peak relative to the peak in fraction 7, suggesting a molecular basis for the increased safety of PAV-621 by virtue of higher affinity for one of the two putative multi-protein complexes targeted by PAV-951 (see figure 5*c*).

## 3. Discussion

Our data indicate that PAV-617 and PAV-951, two structurally unrelated protein assembly modulator antiviral compounds selected by novel phenotypic screens for their ability to arrest proliferation in distinctive ways, are cytotoxic to over 70 neoplastic cell lines representing both rare and common cancers. These compounds, while early in their drug optimization, performed comparably well to the commercial anti-cancer drug Gemcitabine, at 10 and 60 fold lower doses, to inhibit the growth of A549 lung cancer cell line in immunodeficient mice. PAV-951 was also able to reduce growth of a HT-29 colorectal cancer in a mouse xenograft study, though to a lesser degree than the positive control Irinotecan. These data suggest that these two compounds are directed to two different targets common to a wide range of cancers and may provide a starting point for the development of novel cancer therapeutics.

**Figure 7.**
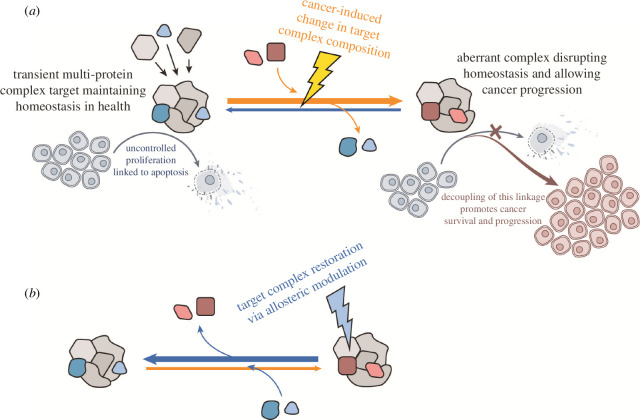
Cartoon diagram of proposed mechanism of action of assembly modulating compounds. The proposed model where a normal multi-protein complex that plays a role mediating the linkage between uncontrolled proliferation and apoptosis is modified into an aberrant multi-protein complex at an early, precancerous stage allowing cancer progression rather than homeostatic elimination. Electronic supplementary material, figure S7B shows the proposed mechanism where treatment with a protein assembly modulating compound restores the original multi-protein complex and its homeostatic functions, including elimination of the cancer. Allosteric modulation is indicated as a means by which these changes may be induced.

DRAC experiments with free drug eluates analysed by sucrose gradient centrifugation indicate that PAV-617 and PAV-951 interact with proteins that are components of multi-protein complexes. These complexes are dynamic, as demonstrated by changes in the eluate when DRAC is carried out in the presence versus absence of metabolic energy substrates or from untreated versus compound-treated cell starting extract. Mass spectrometry of the DRAC eluates reveal the contents to include many proteins whose functions are implicated in the literature as relevant for both cancer and viral interactions. Taken together with other recent work [10,15], these findings suggest a new model for disease pathogenesis in which previously unappreciated transient multi-protein complexes relevant for viral assembly play an additional and important role in the dynamics linking cellular proliferation to apoptosis. We hypothesize that cancer progression is facilitated by aberrant versions of these multi-protein complexes in which the linkage of inappropriate proliferation to apoptosis is attenuated or slowed. The effect of protein assembly modulating compounds may be to restore the original version of the multi-protein complex observed in healthy cells and conforming to the dictates of homeostasis, possibly through an allosteric mechanism-of-action (see figure 7) [36].

There are a plethora of open questions that emerge from these studies, and which the tools developed here should make possible to address. A first question is which of the protein(s) contained in the target complex are directly bound to the compounds and which proteins are indirectly co-associated through protein–protein interactions. Photo-crosslinking has been used previously [37] for identification of direct drug-binding proteins of assembly modulator small molecules [10]. Initial studies by that method here have not yet given a definitive answer. There may be several reasons for this. Most notably, a power of the DRAC methodology is the high density of drug on the resin may allow enrichment of a transient multi-protein complex. Photo-crosslinking which is done at the far lower drug concentrations represented by the EC90 for target engagement, may be unable to detect signal above noise. Moreover the dynamic nature of the target multi-protein complexes which may obscure a transient drug–protein interaction. One way to address these possibilities is continued SAR improvement lowering target binding from the current mid triple digit nanomolar range to single or double digit nanomolar activity, an effort that is currently in progress. This hypothesis is supported by the data showing that the advanced analog PAV-621 (i.e. which is more potent against cancer cells and nevertheless safer in mice) resin eluate is enriched for a subset of the proteins found in the PAV-951 resin eluate (see figures 5 and 6 and electronic supplementary material, figures S7–S9).

A second question is the difference in activity uniquely observed in the PAV-951 series when compound is added to cells at the time of plating, rather than 24 h later (see figure 3*a* and electronic supplementary material, figure S10). The procedure to plate cells involves trypsinization, which detaches adherent cells from the plates on which they are grown, so they can be transferred to a new plate [24]. High expression of trypsin in cancer cells is associated with the epithelial-to-mesenchymal transition (EMT), which leads to metastasis and poor prognosis [35,38–43]. A cell culture model for studying metastasis through differential trypsinization, has noted that trypsin sensitive subpopulations have increased ALDH activity and generate more metastasis than trypsin-resistant subpopulations [35,44]. If a relationship exists between the multi-protein complex target of PAV-951 (which includes several context-dependent metastasis-implicated proteins such as ALDH, CAP1, GSTP1, HSP90, PDI, TKT and VCL, see electronic supplementary material, figure S8) and progression to metastasis, further studies conducted under trypsinized conditions may be provide insights on the relationship between protein assembly and EMT as it relates to the hallmarks of cancer [33,41,45–63].

One phenomenon which we have observed for other, structurally unrelated, assembly modulator chemotypes is that a substantial barrier exists to the development of drug resistance [10,13]. Resistance mutations did not develop against PAV-835, a assembly modulator with anti-influenza activity, after seven passages in the presence of compound [10]. Resistance mutations did not develop against PAV-206, an assembly modulator compound with antiviral activity against HIV, even after 37 serial passages in the presence of compound [13]. Further work is needed to better understand this substantial barrier to resistance development. However, one hypothesis is that this phenomenon may be due to the fundamental importance of assembly pathways in promoting the feedback loops which preventing and/or correcting deviations from homeostasis. Some of the cell lines on which PAV-617 and PAV-951 have displayed efficacy are resistant to other chemotherapies [64]. While this is a promising indication that PAV-617 and PAV-951 might provide a viable treatment option for patients that have not responded to existing therapies, it does not provide information as to whether or not a barrier to the development of resistance will be seen for cancers in the way it is for viruses. The role of ALDH1A1, observed as part of the DRAC-identified multi-protein complex target, in facilitating development of drug resistance in cancer, as described in the literature, may be of relevance when answering this question [32,34].

A final question that requires further study is, if the chemical series based on PAV-617 and/or PAV-951 (whose anti-tumour activities are already on par with existing cancer drugs) is advanced into drugs—how safe will it be for patients? The data here includes two lines of evidence for safety. First, a non-cancer cell line, MRC-5 and a benign reprogrammed stem cell line BB1 by independent assays show strikingly greater safety than cancer lines (see figure 3*d* and electronic supplementary material, figure S6). In particular, at doses of both the early (PAV-951) and advanced analog (PAV-621) that are highly lethal to cancer, no significant activity is observed on the non-cancer lines. Second, despite a dramatic increase in the potency against cancer lines (see figure 6) the safety of advanced analogs in mice is increased.

Animal safety is an important albeit not the only metric for understanding the delta between safety and toxicity. The observed animal toxicity of PAV-617 and PAV-951 is higher than would be ideal for the clinic, but their SAR advancement is a work in progress that is by no means complete. Nevertheless, a handful of FDA-approved cancer drugs have comparable toxicity gauged by mouse MTD—cisplatin has an MTD of 6 mg kg^−1^, Doxorubicin has an MTD of 10 mg kg^−1^ and Vinorelbine has an MTD of 10 mg kg^−1^—and many other cancer drugs are administered to patients despite adverse effects because of the urgency of their condition [65,66]. It is therefore, highly important to note that the doses administered of PAV-617 and PAV-951 in the xenograft studies showed significant inhibition of tumour growth without any serious adverse effects in the animals including clinical signs, weight loss over time, histopathological changes or differences in blood cell count (see figure 4 and electronic supplementary material, figure S7). SAR advancement of the PAV-951 series has led to compounds which have greater lethality against cancer cells at equivalent doses, while also demonstrating improved safety to animals (see figure 6). This improvement to safety cannot be attributable to a lack of exposure since a safe dose of PAV-621 achieves comparable concentrations in lung tissue as PAV-951 (see electronic supplementary material, figure S6F). Rather, it indicates that toxicity to animals is not an inherent component of the anti-cancer mechanism-of-action. These findings suggest that the novel and unconventional transient multi-protein complex target of these compounds may be a truly cancer-selective hallmark. While more needs to be done to demonstrate safety and understand its molecular basis, these early lines of evidence are reassuring.

A biochemical correlation between SAR optimization of safety and efficacy and the changes to the protein components that comprise the target may be key to understanding the roles particular proteins play in maintaining or deviating from homeostasis. Nearly all the proteins identified in the PAV-621 (safer and more efficacious analog of the PAV-951 lead series) resin eluate by MSMS were identified in the PAV-951 resin eluate (see electronic supplementary material, figure S7). However, the converse is not true—less than half of the proteins from the PAV-951 resin eluate were found in PAV-621 resin eluate (see electronic supplementary material, figure S7). This mirrors what we have seen for other, structurally unrelated, assembly modulator compounds where advanced analogues are more selective for their target complexes than their parent compound is [10]. A prediction of this hypothesis is that further optimization of the PAV-951 lead series beyond PAV-621 will yield chemical analogs with even greater increases in both safety and activity as the series becomes more selective for the disease-selective multi-protein complex targets. Studies on a structurally unrelated antiviral assembly modulator for a different therapeutic area [10] suggest the effect of the compound is to restore the aberrant multi-protein complex target to the composition observed in the healthy state. If future studies confirm that finding for the anti-cancer assembly modulators it would suggest that safety derives not only from selectivity for the disease-specific target, but from the action of the compound which is to restore feedback loops of homeostasis whose loss is a hallmark of cancer.

Effective cancer treatments have been developed based on a number of mechanisms including alkylating agents, antimetabolites, antimitotics and monoclonal antibodies [67]. However, as far as we know, no one has attempted to treat cancer through modulation of protein assembly. By identifying two structurally unrelated drug-like small molecules that appear to have distinct and novel targets (transient multi-protein complexes) our work with PAV-617 and PAV-951/PAV-621 suggest the existence of a class of novel targets including for cancer.

It is perhaps not surprising that new methods, such as the approach to drug discovery which we have applied here, and the novel and unconventional targets they allow to be detected, make possible a path to drugs with novel and remarkable properties such as pan-cancer efficacy without healthy cell toxicity. Further work will clarify whether, as we hypothesize, the transience of our targets reflects their involvement as a molecular basis for homeostasis. This conclusion, supported by the consequences of protein assembly modulator treatment both here for cancer and previously for viruses, frames future experiments to better understand this orthogonal approach to development of more physiological disease therapeutics.

## 4. Methods

### 4.1. Lead contact and materials availability

Further information and requests for resources and reagents should be directed to and will be fulfilled by V.R.L. (vlingappa@prosetta.com).

Use of unique compounds PAV-617 and PAV-951 and their stable derivatives may be available upon request by V.R.L. if sought for experimental purposes under a valid completed Materials Transfer Agreement.

The number of replicates carried out for each experiment is described in the figure/table legends.

### 4.2. Chemical synthesis

All compounds synthesized were confirmed by LCMS with purity typically >98%.

#### 4.2.1. Synthesis of PAV-617

See electronic supplementary material, figure S1A.

#### 4.2.1.1. Phenothiazin-5-ium tetraiodide hydrate

Phenothiazin-5-ium tetraiodide hydrate: a solution of phenothiazine (4.98 g, 25 mmol) in anhydrous chloroform (50 ml) was stirred at 50 °C and the solution of iodine (12.7 g, 50 mmol) in CHCl_3_ (250 ml) was added dropwise over 4 h. The resulting dark solution was stirred for an additional 3 h at 50°C, monitored by TLC. After the disappearance of the starting material, the resulting precipitate was filtered, washed with a copious amount of chloroform, dried overnight in vacuo to afford a dark solid (13.9 g, 74%).

#### 4.2.1.2. 3,7-Di(pyrrolidin-1-yl) phenothiazinium iodide

A solution of phenothiazin-5-ium tetraiodide hydrate (2.8 g, 3.6 mmol) in mixture acetonitrile/methanol (50 ml) and pyrrolidine (710 mg, 10 mmol) was stirred for 4 h at room temperature. The resulting mixture was concentrated to dryness and purified by flash chromatography using the methanol–chloroform gradient to purify the desired compound.

#### 4.2.2. Synthesis of PAV-617 resin

See electronic supplementary material, figure S1B.

#### 4.2.2.1. 2-Aminomethylphenothiazine

2-Cyanophenothiazine (2.24 g, 10 mmol) was dissolved in THF (50 mM). Solution of 1 M lithium aluminum hydride (LiAlH_4_) in THF (20 ml, 40 mmol) was added at room temperature and reaction mixture was stirred for 24 h. Mixture was diluted with ether and cooled to 0°C. To this solution water (1.5 ml) (slowly), 15% aqueous sodium hydroxide (1.5 ml) and water (4.5 ml) were added. Mixture was warmed to RT and stirred 15 min. Some anhydrous MgSO4 was added, stirred 15 min, filtered to remove salt. Solution was concentrated and product was used without purification. Yield—1.9 g (83% theor.). M+1 = 229 (LCMS).

#### 4.2.2.2. 2-Bocaminomethylphenothiazine

2-Aminomethylphenothiazine (4.0 g, 17.5 mmol) was dissolved in DCM (100 ml) and Boc-anhydride (3.85 g, 17 .5 mmol) was added with stirring at RT. After 1 h mixture was concentrated and product was purified with flash chromatography (solvent system: hexane–ethyl acetate). Yield—4.0 g (70% theor.). M+1 = 329.

#### 4.2.2.3. 2-Bocaminomethylphenothiazin-5-ium tetraiodide hydrate

Solution of 2-Bocaminophenothiazine (984 mg, 3 mmol) in anhydrous chloroform (10 ml) was stirred at 50°C and the solution of iodine (3.81 g, 15 mmol) in CHCl3 (90 ml) was added dropwise over 4 h. The resulting dark solution was stirred for an additional 3 h at 50°C, monitored by TLC. After the disappearance of the starting material, the resulting precipitate was filtered, washed with a copious amount of chloroform, dried overnight in vacuo to afford a dark solid. Yield—1.5 g (60% theor.).

#### 4.2.2.4. 2-Bocaminomethyl-3,7-di(pyrrolidin-1-yl) phenothiazin-5-ium iodide

A solution of 2-Bocaminomethylphenothiazin-5-ium tetraiodide hydrate (426 mg, 0.5 mmol) in chloroform (10 ml) and pyrrolidine (85 mg, 0.1 ml, 1.2 mmol) was stirred for 1 h at room temperature. The resulting mixture was concentrated to dryness and purified by flash chromatography using the methanol–chloroform gradient to provide the title compound. Yield—207 mg (70% theor.). M = 465 (LCMS).

#### 4.2.2.5. 2-Aminomethyl-3,7-di(pyrrolidin-1-yl) phenothiazin-5-ium dihydrochloride

2-Bocaminomethyl-3,7-di(pyrrolidin-1-yl) phenothiazin-5-ium iodide (590 mg, 1 mmol) was mixed with 4M solution of HCl in 1,4 dioxane (5 ml). Solution was stirred 3 h at room temperature. The resulting mixture was concentrated to dryness and purified by flash chromatography using the methanol–chloroform gradient to provide the title compound. Yield—280 mg (70% theor.). M+1 = 365 (LCMS).

To a solution of affi-gel (Bio-Rad, 10 ml) in a solid phase synthesis tube with frit was added a solution of 2-Bocaminomethyl-3,7-di(pyrrolidin-1-yl) phenothiazine-5-ium iodide (54 mg, 0.11 mmol) and DIEA (1.0 ml) in isopropyl alcohol (4 ml) and the tube was put in a shaker for 12 h. Excess reagents were drained and the resin was washed with isopropyl alcohol (3×) and then saved in isopropyl alcohol.

#### 4.2.3. Synthesis of PAV-951

See electronic supplementary material, figure S2A.

A mixture of 4,7-dichloroquinazoline ((0.811 g (4.1 mmol)) 1 and Isopentylamine (1 g (12 mmol)) in 5 ml of ACN was briefly sonicated and then stirred at room temperature for 20 min and then diluted with EtOAc. The EtOAc solution was then washed with water, dried (Na_2_CO_4)_ and the solvent removed. Crude material was columned with ISCO EtOAc/ CHCl_3_ affording 1 g (97%) of 2.

To a mixture of 7-chloro-*N*-isopentyl-quinazolin-4-amine (1 g (4 mmol)) 2, RuPhosPd (150 mg (0.2 mmol)) , RuPhos 90 mg (0.2 mmol), Cs_2_CO_3_ (3.93 g (10 mmol)) and 20 ml of t-BuOH was added piperazine (172 mg (2 mmol)) . The mixture was heated and stirred at 87°C for 20 h. After cooling to room temp the mix was diluted with 20 ml of sat. NaCl solution and extracted 2× with EtOAc. The extracts were dried (Na2SO_4_) and the solvent removed. The crude was purified with ISCO using 10% NH_3_ in MeOH/CHCl_3_ affording 600 mg (60%) of the dimer PAV-951 [3].

#### 4.2.4. Synthesis of PAV-951 resin

See electronic supplementary material, figure S2B.

A mixture of Bocpiperazine (223 mg(1.2 mmol)), Cs_2_CO_3_ (600 mg (1.5 mmol)), PdRuPhos (37 mg (0.05 mmol)), RuPhos (23 mg (0.05 mmol)) and 7-chloro-*N*-isopentyl-quinazolin4-amine (250 mg (1 mmol)) 2 in 2–3 ml t-BuOH was heated to 87°C with stirring under argon for 20 h. After cooling the mixture was diluted with 20 ml of 10% MeOH/DCM and the resulting mix was filtered through a short pad of celite. The filtrate was rotary evaporated to dryness and the residue was purified on the ISCO (Hexane/EtOAc) affording 384 mg (96%) of the Bocpiperazine adduct 4.

A mixture of 4,7-dichloroquinazoline ((0.189 g (0.95 mmol)) 1 and tert-Butyl 4-(2-aminoethyl)piperidine-1-carboxylate (0.433 g (1.9 mmol)) in 3 ml of ACN was briefly sonicated and then stirred at room temperature for 20 min and then diluted with EtOAc. The EtOAc solution was then washed with water, dried (Na_2_CO_4_) and the solvent removed. Crude material was columned with ISCO EtOAc/CHCl_3_ affording 296 mg (80%) of 5.

To Bocpiperazine adduct (223 mg(1.2 mmol)) 4 was added 3 ml of 30% TFA/DCM and the mixture stirred for 1 h at room temperature. The mixture was then rotary evaporated to dryness and the residue was partition between 1N NaOH and DCM. The DCM layer was dried (Na_2_SO_4_) and the solvent removed affording 228 mg of the Piperazine adduct 6 which was used as is in the next step.

A mixture the piperazine compound (228 mg (0.76 mmol)), Cs_2_CO_3_ (376 mg (1.15 mmol)), PdRuPhos (33 mg (0.04 mmol)), RuPhos (19 mg (0.04 mmol)) and the chloroquinazoline compound (296 mg (0.76 mmol)) 5 in 3 ml t-BuOH was heated with stirring at 87 °C under argon for 20 h. After cooling the mixture was diluted with 20 ml of 10% MeOH/DCM and the resulting mix was filtered through a short pad of celite. The filtrate was rotary evaporated to dryness and the residue was purified on the ISCO 10% NH_3_ in MeOH/CHCl_3_ affording 375 mg (75%) of the desired unsymmetric dimer PAV-504 [7].

To the Dimer 7 (6.5mg (0.01 mmol)) was added 0.5 ml of 4N HCl in Dioxane and the resulting mix was allowed to stir for 30 min at room temperature. The mixture was rotary evaporated to dryness and the residue dissolved in 1.5 ml of isopropanol. Next DIEA (10 µl (0.06 mmol)) and a catalytic amount of DMAP (0.2 mg) was added to the isopropanol solution. The resulting mix was added to affi-gel-10 (1 ml (0.015 mmol)) in a fritted tube and the mixture was agitated on a shaker at room temperature overnight. Excess reagents were then drained and the resin was washed with 3 ml (3×) of isopropanol and then stored in isopropanol affording 1 ml of PAV-951 resin [8].

### 4.3. Experimental models and subject details

#### 4.3.1. Cell lines

The *in vivo* xenograft study utilized A549 (male human lung cancer) and HT-29 (female human colon cancer) cell lines. Cells were grown under sterile conditions. These studies were conducted at Anthem Biosciences Private Limited in Bangalore, India.

The human tumour cell proliferation assay used A172 (male human glioma), BFTC-905 (female human urinary bladder transitional cell carcinoma), COR-L105 (male human lung adenocarcinoma), DB (male human b-cell lymphoma), FaDu (male human pharynx squamous cell carcinoma), H9 (male human t-cell lymphoma), Hs 294T (male human melanoma), MCF7 (female human breast cancer), MDA MB 436 (female human breast cancer), MeWo (male human melanoma), MHH-PREB-1 (male human b-cell lymphoma), SJSA1-OSA (male human osteosarcoma), SU-DHL-10 (male human b-cell lymphoma), SW1353 (female human chondrosarcoma) and U-2 OS (female human osteosarcoma) cell lines. This study was conducted by Eurofins Scientific as part of their OncoPanel™.

The Mpox virus infectious virus assay used BSC-40 cells (nonhuman primate kidney) and Mpox Zaire 79 strain. These studies were conducted by the United States Army Medical Research Institute of Infectious Diseases in Frederick, Maryland.

Human immunodeficiency virus infectious virus assay used MT-2 cells (female human t-cell leukaemia) and the NL4-3 Rluc reporter virus. These studies were conducted at the University of Washington in Seattle, Washington.

The *in vitro* screens for apoptosis, high-density/low-density activity and cell growth recovery utilized the A549, PANC-1, LNCaP C-33 (male human prostate cancer), LNCaP C-81 (male human prostate cancer), CHO K-1 (Chinese hamster ovary) and Hennes 20 (Chinese hamster ovary) cell lines. Cells were grown under sterile conditions. These studies were conducted at Prosetta Biosciences in San Francisco, California.

The *in vitro* drug resin affinity chromatography utilized A549 (male human lung cancer) cell line. Cells were grown under sterile conditions. Sterile conditions were not maintained once cells were harvested for *in vitro* experiments. These studies were conducted at Prosetta Biosciences in San Francisco, California.

#### 4.3.2. BB1 and BB5 adenoma versus carcinoma screen

Two cell lines, BB1 and BB5, were derived from two patient breast biopsies that were obtained prior to treatment. Follow-up classification indicated that BB1 was a benign adenoma and BB5 was a breast adenocarcinoma. The cells were grown in conditional reprogramming media in co-culture with irradiated fibroblasts as described in Liu *et al*. [68]. Once cultures reached exponential growth phase, the cells were harvested and treated with either PAV-617 or PAV-951. These studies were conducted at the Sunnybrook Health Science Center in Toronto, Canada.

#### 4.3.3. National Cancer Institute 60 cell line screen

A panel of cancer cell lines (CCRF-CEM, HL-60 (TB), K-562, MOLT-4, RPMI-8226, SR, A549/ATCC, EKVX, HOP-62, HOP-92, NCI-H226, NCI-H23, NCI-H322M, NCI-H460, NCI-H522, COLO 205, HCC-2998, HCT-116, HCT-15, HT29, KM12, SW-620, SF-268, SF-295, SF-539, SNB-19, SNB-75, U251, LOX IMVI, MALME-3M, M14, MDA-MB-435, SK-MEL-2, SK-MEL-28, SK-MEL-5, UACC-257, UACC-62, IGROV1, OVCAR-3, OVCAR-4, OVCAR-5, OVCAR-8, NCI/ADR-RES, SK-OV-3, 786-0, A498, ACHN, CAKI-1, RXF 393, SN12C, TK-10, PC-3, DU-145, MCF7, MDA-MB-231/ATCC, HS 578T, BT-549, T-47D, MDA-MB-468) were used to assess PAV-617, PAV-951, PAV-621, PAV-541 and PAV-436. This study was conducted at the National Cancer Institute.

### 4.4. Animal models

Maximum tolerated dose (MTD) studies were conducted using female Balb/c mice, aged 8–10 weeks or female CD1 mice, aged 5–6 weeks. Treatment groups were made up of three animals each, unless otherwise noted, and dosing regimens for disclosed data is provided. Animals were sacrificed at the end of the study period using an overdose of CO_2_. MTD studies were conducted at Vipragen Biosciences Private Limited or Radiant Research Services Private Limited in accordance with the Committee for the Purpose of Control and Supervision of Experiments on Animals (CPCSEA) guidelines and Animal Research: Reporting of *in vivo* Experiments (ARRIVE) guidelines.

Pharmacokinetic (PK) studies were conducted in male Sprague Dawley Rats aged 8–10 weeks or male CD1 mice aged 5–6 weeks or Balb/c nude mice. Treatment groups were made up of four animals each and dosing regimens for disclosed data is provided. Animals were sacrificed at the end of the study period using an overdose of CO_2_. PK studies were conducted at Vipragen Biosciences Private Limited or Pharmaron in accordance with the CPCSEA guidelines and ARRIVE guidelines.

Tumour xenograft studies were conducted using female Athymic Nude mice, strain CrTac: Ncr-Foxn1nu, aged 6–8 weeks. Tumour transplantation occurred through subcutaneous injection of a 0.1 ml cell suspension containing 1 to 5 × 10^6^ A549 lung cancer cells obtained from ATCC in Matrigel in PBS into the left flank region of the mice. Treatment groups were made up of six animals each and dosing regimens for disclosed data is provided. Animals were sacrificed at the end of the study period using an overdose of isoflurane anesthesia. Both xenograft studies were conducted at Anthem Biosciences Private Limited in Bangalore, India, and were approved by the Institutional Animal Ethics committee (IAEC) of Anthem Biosciences in accordance with the CPCSEA guidelines and ARRIVE guidelines.

See 'Ethics' section below for approval numbers and the data to which they apply

#### 4.4.1. Biochemical experiments

Drug resin affinity chromatography and SDS–PAGE/silver stain/Western blot analysis of the results, were conducted by Prosetta Biosciences in San Francisco, California, under conditions described in figure legends. Results from disclosed *in vitro* experiments were repeated in triplicate unless otherwise stated. Mass spectrometry analysis of samples were conducted by MS Bioworks in Ann Arbor, Michigan.

### 4.5. Method and analysis details

#### 4.5.1. Mpox virus infection assay

BSC-40 cells of 95% confluence in 24-well plates were infected with 100 pfu of Mpox Zaire 79 diluted in Eagle’s Minimum Essential Medium with 2% foetal bovine serum and incubated in 37°C in 5% CO2 for 1 h. The viral inocula were removed and replaced with the test compounds in six half log dilutions (0.1 ml per well) and the cells were overlaid with 1% methylcellulose in growth media (1 ml per well). The media and virus control cells received growth medium containing 1% methylcellulose. After 3 days of infection, when plaques appeared, cells were stained with crystal violet for an hour and then washed with water and dried overnight. The plaques were counted the next day and virus-only wells were compared with the compound-added wells to determine percentage protection. Infected cells were stained with crystal violet and viral plaques were counted. Averages and standard deviation for plaques observed under different treatment conditions were calculated in Microsoft Excel and graphed as the percent inhibition in PAV-617 treated cells compared with untreated cells.

#### 4.5.2. Human immunodeficiency virus infectious virus assay

MT-2 cells were pre-seeded in 96-well plates in 100 µl of complete RPMI. Multiple concentrations of PAV-951 were serially diluted in DMSO then into an infection media prepared by diluting NL4-3 Rluc virus stock to 400 IU/100 µl with complete RPMI, which was transferred onto the MT-2 cells with a final MOI of 0.02 and final DMSO concentration of 1% in infected places. One well received DMSO only, instead of PAV-951, and one well received medium only for normalization and background collection. Cells were incubated at 37°C for 96 h. A 100 µl of medium was removed and discarded and 10 µl of 15 µM EnduRen luciferase substrate was added to each well, followed by incubation for 1.5 h at 37 °C. Plates were read on a luminescence plate reader. Bioluminescence intensity was read on a Synergy H1 BioTek plate reader. Averages and standard deviation for viral titer observed under different treatment conditions were calculated in Microsoft Excel and graphed as the percent inhibition in PAV-951 treated cells compared with untreated cells.

#### 4.5.3. Apoptosis screen

A 96-well plate was seeded with Hennes 20 cells at 500 cells per well, CHO K-1 cells at 500 cells per well, LNCaP C-33 cells at 2000 cells per well, and LNCaP C-81 cells at 2000 cells per well. Cells were grown in 100 µl minimum essential media for 3 days then three wells of each cell line received treatment with 1% DMSO. Twelve hours after drug treatment, a mixture of 25 µl media and 25 µl Apo-ONE reagent (Promega) was added then the plate was covered and placed on a shaker at room temperature for 6 h. The plate was read on a microplate reader for fluorescence at 499/521. Values were averaged and standard deviations were calculated for each triplicate condition and graphed on Microsoft Excel.

#### 4.5.4. High density/low density assay

Two 96-well plates were seeded with Hennes 20 cells in parallel where one was plated at a density of 500 cells per well and the other was plated at a density of 15 000 cells per well. 90 µl of minimum essential media was added to each well and plates were placed in a 37 °C incubator for 24 h. The next day, 10 µl of media containing dilutions of compound in DMSO were added to each plate in triplicate with final concentrations of 0.025 µM PAV-617, 0.05 µM PAV-617, 0.1 µM PAV-617, 0.5 µM PAV-617, 0.02 µM PAV-951, 0.3 µM PAV-951, 0.4 µM PAV-951 or 0.5 µM PAV-951. Six wells on each plate received 10 µl of media containing only DMSO. Each well was gently mixed five times with a 100 µl pipette. Plates were incubated at 37°C for 72 h then 10 µl of AlamarBlue was added to each well. Wells were mixed five times then incubated at 37°C for 72 h. Plates were then read at 530/590. Values were averaged and standard deviations were calculated for each triplicate condition and graphed on Microsoft Excel.

#### 4.5.5. Cell growth recovery assay

A 96-well plate was seeded with either Hennes 20 or LNCaP C-33 cells at 500 cells per well in 90 µl of minimum essential media and incubated at 37°C for 24 h. 0.5% DMSO was diluted in media and added to six control wells for each plate. PAV-617 was diluted in media and added to three wells at a concentration of 0.3 µM. PAV-951 was diluted in media and added to concentration of 0.4 µM. After 24 h of PAV-617 treatment or 6 h of PAV-951 treatment, the medium containing compound was removed and replaced with fresh media. After 72 h (day 5), plates were assayed with AlamarBlue and fluorescence was read at 530/590. The medium containing AlamarBlue was removed and replaced with fresh media. After another 72 h (day 8) plates were assayed with AlamarBlue and fluorescence again, then medium containing AlamarBlue was removed and replaced with fresh media. After a final 72 h incubation (day 11), plates were assayed with AlamarBlue one more time. Average fluorescence for each day and treatment condition was graphed on Prism and IC50s were calculated with the nonlinear regression (Inhibitor) versus normalized response—variable slope.

#### 4.5.6. Human tumour cell proliferation assay

A panel of human tumour cell lines (A172, BFTC-905, COR-L105, DB, FaDu, H9, Hs 294T, MCF7, MDA MB 436, MeWo, MHH-PREB-1, SJSA1-OSA, SW1353 and U2OS) were grown in RPMI 1640, 10% FBS, 2 mM L-alanyl-L-glutamine, 1 mM Na pyruvate. Cells were seeded into 384-well plates and incubated in a humidified atmosphere with 5% CO_2_ at 37°C. After 24 h of incubation DMSO, PAV-617 or PAV-951 was added at concentrations of 5 µM, 1 µM and 0.2 µM and plates were incubated for 3 days. Then cells were lysed with CellTiter-Glo (Promega) which generates a bioluminescence signal relative to ATP levels and is used as a measurement of viable cells. Bioluminescence was read by a PerkinElmer Envision microplate reader. Bioluminescence intensity was measured by a PerkinElmer Envision microplate reader and transformed to a percent of control (POC) using the formula: POC=(Ix/I0) × 100, where Ix is the whole well signal intensity at a given treatment, and I0 is the average intensity of the untreated vehicle wells. Data was graphed on Prism and IC50s were calculated with the nonlinear regression (inhibitor) versus normalized response—variable slope.

#### 4.5.7. BB1 and BB5 adenoma versus carcinoma screen

Two cell lines, BB1 and BB5, were derived from two patient breast biopsies that were obtained prior to treatment. Follow up classification indicated that BB1 was a benign adenoma and BB5 was a breast adenocarcinoma. The cells were grown in conditional reprogramming media in co-culture with irradiated fibroblasts as described in Liu *et al*. [68]. Once cultures reached exponential growth phase, the cells were harvested and seeded. After 48 h of growth, cells were treated with PAV-617, PAV-951, 0.1% DMSO (vehicle) or 1 µM staurosporine (positive control which induced cell death) for 60 h. Compound treated cells were imaged with Hoechst 33342, TMRE and MultiChrome-3 which stained the nucleus, functional mitochondria and cellular membranes, respectively.

#### 4.5.8. National Cancer Institute 60 cell line screen

In the NCI-60 screen, 59 cancer cell lines (CCRF-CEM, HL-60 (TB), K-562, MOLT-4, RPMI-8226, SR, A549/ATCC, EKVX, HOP-62, HOP-92, NCI-H226, NCI-H23, NCI-H322M, NCI-H460, NCI-H522, COLO 205, HCC-2998, HCT-116, HCT-15, HT29, KM12, SW-620, SF-268, SF-295, SF-539, SNB-19, SNB-75, U251, LOX IMVI, MALME-3M, M14, MDA-MB-435, SK-MEL-2, SK-MEL-8, SK-MEL−5- UACC-257, UACC-62, IGROV1, OVCAR-3, OVCAR-4, OVCAR-5, OVCAR-8, NCI/ADR-RES, SK-OV-3, 786-0, A498, ACHN, CAKI-1, RXF 393, SN12C, TK-10, PC-3, DU-145, MCF7, MDA-MB-231/ATCC, HS 578T, BT-549, T-47D, MDA-MB-468) were grown for 24 h then treated with vehicle or compound, or fixed *in situ* with TCA, to represent a measurement of the cell population for each cell line at the time of drug addition. The treated cells were grown for an additional 48 h before being fixed *in situ* with TCA. Fixed cells were then stained with Sulforhodamine B. Absorbance was read to determine cell viability of compound-treated cells relative to both the time at which treatment began and to untreated cells at the end of the study. The NCI-60 screen was initially conducted at a single dose of 2.5 µM, but was subsequently repeated with a dose-titration.

The human tumour cell lines of the cancer screening panel are grown in RPMI 1640 medium containing 5% foetal bovine serum and 2 mM L-glutamine. For a typical screening experiment, cells are inoculated into 96-well microtiter plates in 100 μl at plating densities ranging from 5000 to 40 000 cells per well depending on the doubling time of individual cell lines. After cell inoculation, the microtiter plates are incubated at 37°C, 5% CO_2_, 95% air and 100% relative humidity for 24 h prior to addition of experimental drugs.

After 24 h, two plates of each cell line are fixed *in situ* with TCA, to represent a measurement of the cell population for each cell line at the time of drug addition (Tz). Experimental drugs are solubilized in dimethyl sulfoxide at 400 fold the desired final maximum test concentration and stored frozen prior to use. At the time of drug addition, an aliquot of frozen concentrate is thawed and diluted to twice the desired final maximum test concentration with complete medium containing 50 μg ml^−1^ gentamicin.

Following drug addition, the plates are incubated for an additional 48 h at 37 °C, 5% CO2, 95% air and 100% relative humidity. For adherent cells, the assay is terminated by the addition of cold TCA. Cells are fixed *in situ* by the gentle addition of 50 μl of cold 50% (w/v) TCA (final concentration, 10% TCA) and incubated for 60 min at 4°C. The supernatant is discarded, and the plates are washed five times with tap water and air dried. Sulforhodamine B (SRB) solution (100 μl) at 0.4% (w/v) in 1% acetic acid is added to each well, and plates are incubated for 10 min at room temperature. After staining, unbound dye is removed by washing five times with 1% acetic acid and the plates are air dried. Bound stain is subsequently solubilized with 10 mM trizma base, and the absorbance is read on an automated plate reader at a wavelength of 515 nm. For suspension cells, the methodology is the same except that the assay is terminated by fixing settled cells at the bottom of the wells by gently adding 50 μl of 80% TCA (final concentration, 16% TCA).

Using the seven absorbance measurements (time zero, (Tz), control growth, (C), and test growth in the presence of drug at the five concentration levels (Ti)), the percentage growth is calculated at each of the drug concentrations levels. Percentage growth is calculated as:


$$\begin{array}{l} {}[(Ti-Tz)/(C-Tz)]\times 100\;for\;concentrations\;for\;which\;Ti>/=Tz\\ {}[(Ti-Tz)/Tz]\times 100\;for\;concentrations\;for\;which\;Ti<Tz. \end{array}$$

Three dose response parameters are calculated for each experimental agent. Growth inhibition of 50% (GI50) is calculated from [(Ti−Tz)/(C−Tz)] × 100 = 50, which is the drug concentration resulting in a 50% reduction in the net protein increase (as measured by SRB staining) in control cells during the drug incubation. The drug concentration resulting in total growth inhibition (TGI) is calculated from Ti = Tz. The LC50 (concentration of drug resulting in a 50% reduction in the measured protein at the end of the drug treatment as compared with that at the beginning) indicating a net loss of cells following treatment is calculated from [(Ti−Tz)/Tz] × 100 = −50. Values are calculated for each of these three parameters if the level of activity is reached; however, if the effect is not reached or is exceeded, the value for that parameter is expressed as greater or less than the maximum or minimum concentration tested.

#### 4.5.9. Mouse maximum tolerated dose determination

For the intraperitoneal MTD study, female Balb/c mice aged 8–10 weeks were randomly divided into treatment groups with three animals per group. Animals in each treatment group were weighed and received one IP injection of 0.1–0.15 ml containing either vehicle (10% DMSO, 45% propylene glycol, 45% sterile water), 1 mg kg^−1^ PAV-617, 2 mg kg^−1^ PAV-617, 5 mg kg^−1^ PAV-617, 10 mg kg^−1^ PAV−617, 1 mg kg^−1^ PAV-951, 2.5 mg kg^−1^ PAV-951, 5 mg kg^−1^ PAV-951 or 10 mg kg^−1^ PAV-951. Animals were observed from day 0 until day 3 for clinical signs of toxicity. Animals were euthanized after 72 h and were examined externally and internally by a pathologist for abnormalities in organ weight and tissue damage. Blood samples were sent for a complete blood count bioanalysis. MTD was determined to be the dose at which no signs of toxicity were observed by any parameters.

For the oral MTD study, female CD1 mice aged 5–6 weeks were given either an oral dose of vehicle (10% DMSO, 45% propylene glycol, 45% sterile water) or either 10 mg kg^−1^ or 20 mg kg^−1^ of PAV-617 or PAV-951. The vehicle and 20 mg kg^−1^ groups had three animals each, while the 10 mg kg^−1^ groups only had one animal. Animals were observed for clinical signs and after a week they were euthanized and examined externally and internally by a pathologist for changes related to toxicity.

#### 4.5.10. Pharmacokinetics studies

Male Sprague Dawley rats aged 8–10 weeks were randomly divided into treatment groups with four animals per group. Animals in each treatment group were weighed and received one 2.4 ml intravenous dose of either vehicle (10% DMSO, 45% propylene glycol, 45% sterile water), 1 mg kg^−1^ PAV-617, or 0.5 mg kg^−1^ PAV-951, or one intraperitoneal dose of either vehicle (100% labrasol), 5 mg kg^−1^ PAV-617 or 2.5 mg kg^−1^ PAV-951 . Blood was collected from a pre-cannulated line before dosing, and subsequently 15 min, 30 min, 1 h, 2 h, 4 h, 8 h, 12 h, 24 h and 30 h post-dosing. Concentration of drug in the plasma over time was measured using a Waters Acquity TQD LCMS/MS. Maximum concentration (Cmax) was determined to be the maximum concentration detected in a dataset. Half life, area under the curve and mean residence time were calculated with Phoenix WinNolin software.

CD1 or Balb/c nude mice aged 6–8 weeks were randomly divided into treatment groups with three animals per group. Animals in each treatment group were weighed and received either one 2.4 ml intravenous dose of either vehicle (10% DMSO, 45% propylene glycol, 45% sterile water) or 2.5 mg kg^−1^ PAV-951, or one intraperitoneal dose of either vehicle (100% 10% DMSO, 45% propylene glycol, 45% sterile water) or 5 mg kg^−1^ PAV-621. Animals were sacrificed after 1 h, 4 h, 8 h, 24 h post-dosing. Concentration of drug in the plasma and lung over time was measured using a Waters Acquity TQD LCMS/MS. Maximum concentration (Cmax) was determined to be the maximum concentration detected in a dataset. Half life, area under the curve and mean residence time were calculated with Phoenix WinNolin software.

#### 4.5.11. A549 xenograft studies

A549 cells growing in RPMI-1640 medium were suspended with Matrigel in PBS. 0.1 ml of cell suspension containing 1 × 10^6^ cells were injected subcutaneously into the left flank region of female, 6–8 weeks old nude mice (CrTac: Ncr-Foxn1nu). After 30 days of tumour establishment, mice were divided randomly into treatment groups. In the PAV-617 study, six animals were treated with vehicle only (10% DMSO, 10% propylene glycol, 80% sterile water) by IP once daily, six animals were treated with 100 mg kg^−1^ Gemcitabine hydrochloride by IP twice weekly and six animals were treated with 10 mg kg^−1^ PAV-617 by IP once daily for 28 days. In the PAV-951 study, six animals were treated with vehicle only (10% DMSO, 10% propylene glycol, 80% sterile water) by IV once daily, six animals were treated with 100 mg kg^−1^ Gemcitabine by IV twice weekly, and six animals were treated with 1.5 mg kg^−1^ PA-951 by IV once daily for 14 days. In both studies, mice were weighed and their tumours were measured using a digital Vernier caliper. Tumour volume was calculated using the formula: (*L* × *W*2)/2 where *L* is the largest diameter and *W* is the smallest diameter of the tumour. Statistical analysis was performed using GraphPad Prism (Ver. 5.03). Statistical analysis of tumour growth inhibition between the Control and Treated groups was performed by using one-way ANOVA followed by Dunnett’s test.

#### 4.5.12. HT-29 xenograft study

HT-29 cells growing in HBSS medium were suspended in Matrigel. 0.1 ml of the cell suspension containing 5 × 10^6^ cells were injected subcutaneously into the left flank region of male, 6–8 week old SCID mice. After tumour establishment, animals were divided randomly into groups of six and treated daily by IV with vehicle daily for 17 days, 3 mg kg^−1^ PAV-951 three times per week for 17 days, or treated by IP with 60 mg kg^−1^ Irinotecan every 4 days for 14 days. Tumour volume was calculated using the formula: (*L* × *W*2)/2 where *L* is the largest diameter and *W* is the smallest diameter of the tumour. Statistical analysis was performed using GraphPad Prism (Ver. 5.03). Statistical analysis of tumour growth inhibition between the Control and Treated groups was performed by using ne-way ANOVA followed by Dunnett’s test.

#### 4.5.13. Drug resin affinity chromatography

A549 cells were grown in minimum essential media (UCSF) with 10% FBS and 1% Penstrep for 24 h then treated with 500 nM PAV-617, 500 nM PAV-951 or DMSO for 22 h. Cells were scraped into cold phosphate buffered saline (PBS) (10 mM sodium phosphate, 150 mM sodium chloride pH 7.4), then spun at 1000 rpm for 10 min until pelleted. The PBS was decanted and the pellet resuspended in a low salt buffer (10 mM HEPES pH 7.6, 10 mM NaCl, 1 mM MgAc with 0.35% Tritonx100) then centrifuged at 10 000 rpm for 10 min at 4°C. The post-mitochondrial supernatant was removed and adjusted to a concentration of approximately 5 mg ml^−1^ unless otherwise stated and equilibrated in a physiologic column buffer (50 mM Hepes pH 7.6, 100 mM KAc, 6 mM MgAc, 1 mM EDTA, 4 mM TGA). In some conditions, the extract was supplemented with an energy cocktail (to a final concentration of 1 mM rATP, 1 mM rGTP, 1 mM rCTP, 1 mM rUTP, creatine phosphate and 5 µg ml^−1^ creatine kinase). 30 µl of extract was then incubated for 1 h at either 4°C or 22°C on 30 µl of affi-gel resin coupled to either PAV-617, methylene blue, PAV-951, PAV-621 or a 4% agarose matrix (control). The input material was collected and the resin was then washed with 3 ml column buffer. The resins were eluted overnight at either °C or at 22°C in 100 µl column buffer containing either 100 µM PAV-617, 100 µM PAV-951, 100 µM PAV-621 or 1% DMSO, with or without the energy cocktail. In some experiments, the depleted flow through from the resins were incubated on a second column, washed 100× in column buffer, and then stripped with 1% SDS. Eluates were run on SDS–PAGE with samples for silver stain and/or western blot or sent for mass spectrometry analysis.

#### 4.5.14. Sucrose step gradients

Sucrose step gradients were performed as previously described, however due to the inclusion of metabolic energy in the eluates, the gradients were centrifuged at room temperature rather than 4°C [37].

#### 4.5.15. Silver stain

SDS–PAGE gels were incubated overnight in a fixative (50% methanol, 10% acetic acid, 40% water), then for an hour in 50% methanol (done as two washes), and an hour in water (done as two washes). The gels were sensitized in 0.02% sodium thiosulfate for 1 min then washed twice for 30 s with water. The gels were incubated for 30 min in cold 0.1% silver nitrate with 0.02% formaldehyde then washed twice for 30 s. The gels were developed in 3% sodium carbonate with 0.02% formaldehyde. The developed gels showing the pattern of protein bands was scanned and the image was analysed.

#### 4.5.16. Western blotting

SDS–PAGE gels were transferred in Towbin buffer (25 mM Tris, 192 mM glycine, 20% w/v methanol) to polyvinylidene fluoride membrane, blocked in 1% bovine serum albumin (BSA) in PBS, incubated overnight at 4°C in a 1 : 1000 dilution of 100 µg ml^−1^ affinity-purified primary IGG to KAP-1, MTHFD1, hnrnpK, TUBB or PDI in 1% BSA in PBS containing 0.1% Tween-20 (PBST). Membranes were then washed twice in PBST and incubated for 2 h at room temperature in a 1 : 5000 dilution of secondary anti-rabbit or anti-mouse antibody coupled to alkaline phosphatase in PBST. Membranes were washed two more times in PBST then incubated in a developer solution prepared from 100 µl of 7.5 mg ml^−1^ 5-bromo-4-chloro-3-indolyl phosphate dissolved in 60% dimethyl formamide (DMF) in water and 100 µl of 15 mg ml^−1^ nitro blue tetrazolium dissolved in 70% DMF in water, adjusted to 50 ml with 0.1 Tris (pH 9.5) and 0.1 mM magnesium chloride. Membranes were scanned and the integrated density of protein band was measured on ImageJ. Averages and the standard deviation between repeated experiments were calculated and plotted on Microsoft Excel.

#### 4.5.17. Tandem mass spectrometry

Samples were processed by SDS–PAGE using a 10% Bis-tris NuPAGE gel with the MES buffer system. The mobility region was excised and washed with 25 mM ammonium bicarbonate followed by 15 mM acetonitrile. Samples were reduced with 10 mM dithiothreitol and 60°C followed by alkylation with 5o mM iodoacetamide at room temperature. Samples were then digested with trypsin (Promega) overnight (18 h) at 37°C then quenched with formic acid and desalted using an Empore SD plate. Half of each digested sample was analysed by LC-MS/MS with a Waters NanoAcquity HPLC system interfaced to a ThermoFisher Q Exactive. Peptides were loaded on a trapping column and eluted over a 75 µM analytical column at 350 nl/min packed with Luna C18 resin (Phenomenex). The mass spectrometer was operated in a data dependent mode, with the Oribtrap operating at 60 000 FWHM and 15 000 FWHM for MS and MS/MS, respectively. The 15 most abundant ions were selected for MS/MS.

Data was searched using a local copy of Mascot (Matrix Science) with the following parameters: Enzyme: Trypzin/P; Database: SwissProt Human (concatenated forward and reverse plus common contaminants); Fixed modification: Carbamidomethyl (C) Variable modifications: Oxidation (M), Acetyl (N-term), Pyro-Glu (N-term Q), Deamidation (N/Q) Mass values: Monoisotopic; Peptide Mass Tolerance: 10 ppm; Fragment Mass Tolerance: 0.02 Da; Max Missed Cleavages: 2. The data was analysed by label free quantitation (LFQ) and spectral count methods. LFQ intensity values of each condition were measured in triplicate and compared against each other to generate log2 fold change values for each combination of conditions. Spectral counts were filtered for a 1% protein/peptide false discovery rate requiring two unique peptides per protein and the dataset was further adjusted by subtraction of spectral counts for specific proteins observed in the control resin. Identified proteins were searched in the Bushman labs oncogene database (http://www.bushmanlab.org/links/genelists) or the database as described in Jager *et al*. [30] to determine if they interact with HIV.

## Data Availability

Source data can be viewed at the Dryad repository [69]. Supplementary material is available online [70].
